# New Aspects in the Differential Diagnosis and Therapy of Bladder Pain Syndrome/Interstitial Cystitis

**DOI:** 10.1155/2011/639479

**Published:** 2011-10-19

**Authors:** Jochen Neuhaus, Thilo Schwalenberg, Lars-Christian Horn, Henry Alexander, Jens-Uwe Stolzenburg

**Affiliations:** ^1^Department of Urology, University Hospital Leipzig, 04103 Leipzig, Germany; ^2^Department of Pathology, University of Leipzig, 04103 Leipzig, Germany; ^3^Department of Gynecology and Reproduction, University Hospital Leipzig, 04103 Leipzig, Germany

## Abstract

Diagnosis of bladder pain syndrome/interstitial cystitis (BPS/IC) is presently based on mainly clinical symptoms. BPS/IC can be considered as a worst-case scenario of bladder overactivity of unknown origin, including bladder pain. Usually, patients are partially or completely resistant to anticholinergic therapy, and therapeutical options are especially restricted in case of BPS/IC. Therefore, early detection of patients prone to develop BPS/IC symptoms is essential for successful therapy. We propose extended diagnostics including molecular markers. Differential diagnosis should be based on three diagnostical “columns”: (i) clinical diagnostics, (ii) histopathology, and (iii) molecular diagnostics. Analysis of molecular alterations of receptor expression in detrusor smooth muscle cells and urothelial integrity is necessary to develop patient-tailored therapeutical concepts. Although more research is needed to elucidate the pathomechanisms involved, extended BPS/IC diagnostics could already be integrated into routine patient care, allowing evidence-based pharmacotherapy of patients with idiopathic bladder overactivity and BPS/IC.

## 1. Introduction

**Figure 1:**
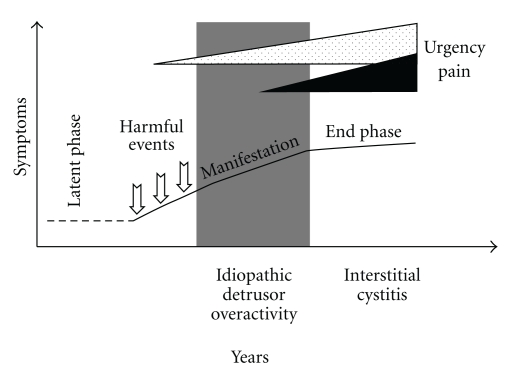
Hypothetical course of BPS/IC development. While urgency develops in early “manifestation” phase, pain symptoms become evident only in late “end” phase, defining full-blown BPS/IC.

There is an ongoing lively discussion about the diagnosis of interstitial cystitis (IC). Diagnosis mainly relies on clinical symptoms, since it has been shown that the more restrictive definition of the National Institute of Diabetes, Digestive, and Kidney Diseases (NIDDK) [1] failed to detect about 60% of the clinically significant IC patients [2]. Recently, IC has been redefined by the European Society for the Study of Interstitial Cystitis (ESSIC), which felt that bladder pain or discomfort to be most important criterion for differential diagnosis and inaugurated the term bladder pain syndrome/interstitial cystitis (BPS/IC) [3]. However, a number of alterations within the bladder wall, regarding detrusor smooth muscle cells [4–7], suburothelial myofibroblasts [8–10], innervation [11–14], urothelial function and integrity [15–19], and cytokine expression [20, 21], have been described, implying that pain symptoms develop relatively late in the cause of the disease. We hypothesize that initial urothelial impairment (unknown origin) initiates a pathophysiological cascade leading in long-term to the development of BPS/IC, and that severe pain symptoms are only present in late phase, that is, full blown clinical picture (Figure 1). 

If patients could be detected at an early stage of the disease, the chance of successful therapeutical intervention would improve. Therefore, we examined patients showing clinical symptoms of BPS/IC to find a pattern of alterations associated with BPS/IC. 

Since the whole bladder wall seems to be involved in bladder dysfunction, it is necessary to evaluate urothelial integrity, detrusor smooth muscle cell receptor expression, alterations in the lamina propria, and afferent nervous control. We here propose a diagnostic approach integrating three diagnostic “columns”, (i) clinical diagnosis, (ii) histopathology, and (iii) molecular diagnostics.

## 2. Materials and Methods

The study was approved by the local Ethics Committee of the University of Leipzig and followed the recommendations of the Helsinki declaration (1964). 

Female patients from our hospital were included into a preliminary study of receptor expression analysis; BPS/IC: *n* = 19; age 61.95 (3.164) years, mean (SEM); ESSIC classification: 2A (0), 2B (4), 2C (8), 2X (7); control: *n* = 9; age 63.19 (3.019) years; female patients undergoing cystectomy due to bladder carcinoma or gynecological tumors. In a second study, we compared the expression of human chorionic gonadotropin; control: *n* = 5; age 62.00 (4.615) years; BPS/IC: *n* = 10; age 59.50 (1.881) years; ESSIC classification CX (4), 2A (1), 2B (1), 2C (2), and 2X (2).

We used confocal immunofluorescence analysis to quantify the expression of muscarinic (M2, M3), purinergic (P2X1, P2X2, P2X3), histamine (H1, H2) receptors, and HCG-beta (Table 1) and used SYBR-green quantitative real-time PCR to examine receptor gene expression (Table 2). Confocal images were acquired at a Pascal 5 laser scanning microscope equipped with a 63 × 1.4 na oil immersion objective (Zeiss, Jena, Germany). Analyses were done using self written ImageJ [22] scripts, OpenOffice (http://www.OpenOffice.org/), and GraphPad Prism version 5 for Mac OS X (GraphPad Software, San Diego, Calif, USA, http://www.graphpad.com/) was used for statistics.

## 3. Results and Discussion

### 3.1. Symptoms of Bladder Pain Syndrome/Interstitial Cystitis (BPS/IC)

Clinically, frequency, urgency, and bladder pain are characteristic for patients suffering from IC. In the early stages of the disease, urgency seems to be the most impressive symptom, and bladder pain develops in later phase, probably in conjunction with advanced urothelial damage and perineuronal inflammation (Figure 1). In the “latent” and early “manifestation” phase, it is essential to distinguish motoric detrusor overactivity from developing BPS/IC. Urodynamics can help to perform differential diagnosis; however, urgency reported by the patient does not always correlate with urodynamical findings. Therefore, we suspect a significant part of the patients diagnosed with OAB syndrome to suffer from an early stage of BPS/IC.

An indirect proof for the significance of urothelial damage for the pathophysiology of the OAB syndrome comes from a study showing higher rate of symptoms improvement after additional application of chondroitin sulfate than by anticholinergic therapy alone [23]. Chondroitin sulfate is instilled into the bladder to restore the glycosaminoglycan (GAG) layer of the urothelium, which is part of the protective urine-tissue barrier of the intact urothelial lining [19]. Therefore, urothelial lesion seems to be the best candidate event for initialization of chronic abacterial cystitis. In case of therapeutical failure of anticholinergics, differential diagnostics of BPS/IC should be performed.

### 3.2. The Three “Columns” of IC-Diagnostics

We feel that it is essential to early include histopathological and molecular biological examination of bladder biopsies into the diagnostic regime. Since all cellular components of the bladder seem to be involved in pathophysiology of BPS/IC, only histological examination of deep biopsy, spanning the whole bladder wall, ensures proper diagnosis.

**(i) Cystoscopy**

**Figure 2:**
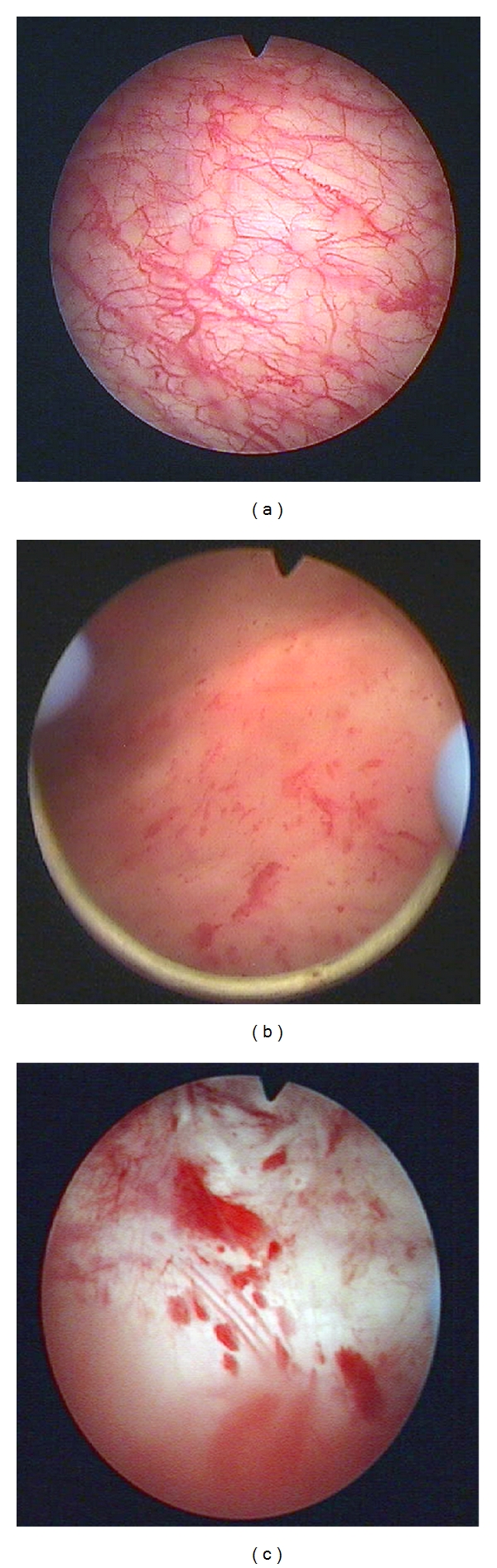
Cystoscopic images of patients showing identical BPS/IC symptoms. Note the heterogeneity of the appearance of the mucosa and the differences in vascularization. (a) Hypervascularization of the bladder wall. (b) Atrophic bladder wall with petechial bleedings after bladder distension. (c) Ulcerative form of BPS/IC.

Despite common patient symptom report, frequency, urgency, and bladder pain, the cystoscopic picture of the bladder might vary considerably (Figure 2). Only in two cases, cystoscopic evaluation would support BPS/IC diagnosis (Figures 2(b) and 2(c)), while the other bladder shows hypervascularization (Figure 2(a)).

Despite this heterogenic cystoscopic appearance, the endoscopic evaluation of the bladder mucosa including the vascularization status reveals valuable information. Therefore, cystoscopy should be included into the diagnostic routine for OAB diagnostics. The description of a pathological cystoscopic findings is essential for early therapeutical conception before pain becomes the dominant symptom of a uncontrollable disease. During this stage of disease often, a clinically hard-to-define urgency component dominates, which is, therefore, referred to as “idiopathic urgency”.

Due to the fact that BPS/IC is mostly regarded as pain syndrome and not as a disease associated with the end organ urinary bladder, cystoscopic diagnostics and bladder provocation tests, which are able to detect defects in the urothelial layer, have come out of focus recently. Therefore, in the next chapters, we will discuss the relevance of histopathological alterations within the bladder wall for the differential diagnosis of BPS/IC. There are two contrary diagnostic approaches, the strategy to pure clinical diagnostics and the concept to evaluate pathological alterations in the different functional units of the bladder wall, which requires, however, invasive diagnostics.

**(ii) Histopathology**

Histopathological examination should be obligatory to enable exclusion of carcinoma in situ (cis). There is no common histomorphological appearance of IC [24–26]. In the early stage of disease, the urothelial lining might be normal, and mastocytosis of the detrusor, initially used to define BPS/IC [2, 27], might be absent even in late, full-blown BPS/IC. An early-stage nonulcerative form (i.e., Figures 2(a) and 2(b)) can be discerned from a late-stage ulcerative form (Figure 2(c)). Histopathological findings supporting the diagnosis of BPS/ICare the following.

1. Urothelial lesions may be present as loss of covering umbrella cells, urothelial flattening, or urothelial denudation. The characteristic finding of urothelial cracking after hydrodistention can be ascribed to those histopathological alterations of the urothelial covering of the bladder wall.
2. Fibrosis of the mucosa, often reaching the muscular layer is found especially in late stages of BPS/IC and accounts for reduced bladder capacity. The fibrosis proceeds with the progression of the disease and is related to chronic inflammation of the bladder wall.
3. Interstitial chronic lymphoplasmacellular infiltration of the lamina propria is a characteristic finding in most of the patients. In addition, about 70% of the BPS/IC patients show perineuronal inflammation [26, 28, 29]. Especially at later stages of the disease, inflammatory infiltrates are also present within the detrusor muscle. However, mastocytosis of the mucosa and the detrusor muscle, which has been regarded as BPS/IC-specific feature [30, 31], seems to be not necessarily associated with BPS/IC [2, 24, 32].
4. Nerve fiber proliferation in the lamina propria and the detrusor muscle is another common histopathological finding in BPS/IC and is regarded as a major neuropathological factor [11, 33, 34]. However, despite neural upregulation contributes to the pathophysiology of BPS/IC, it is still unclear whether it is a causative factor of BPS/IC or an after-effect.
5. Hypovascularization of the urothelial layer has been described [35] and may account for impaired bladder perfusion [36, 37] along with wall thickening of small blood vessels in the submucosa and edematous alterations.

**(iii) Molecular Diagnostics**

Few studies examined regulation of neurotransmitter receptors in the normal and diseased human bladder. Alterations of receptor expression on various cells of the bladder wall including nerve fibers have been demonstrated in OAB [38, 39], idiopathic detrusor overactivity (IDO) [4, 34, 40], neurogenic bladder [41], and interstitial cystitis [5, 12].

**Figure 3:**
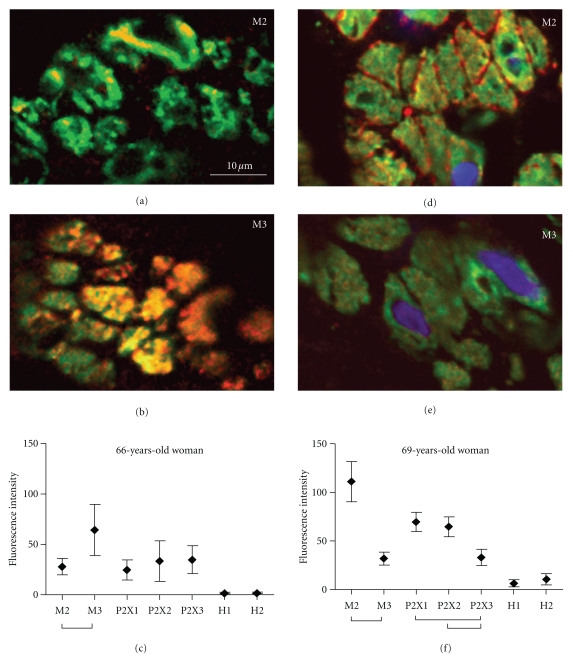
Examples of individual receptor expression profiles based on confocal immunofluorescence analysis. (a–c) showing a female patient with overexpression of M3, P2X1-3 receptors; (d–f) typical distribution in IC patients with overexpression of M2 receptor; in addition, this patient shows high levels of purinergic receptors and significant expression of histamine H2 receptor; aSMCA (green); receptor staining (red); nuclear staining (blue); bar in (a) applies to all micrographs; (c, f) mean ± SD; bars indicate significant differences (ANOVA, Tukey's Test, *P* < 0.05).

Since muscarinic receptors on detrusor smooth muscle cells are the classical target for anticholinergic therapy, we included the expression of M2 and M3 subtypes in the receptor analysis of the detrusor. In addition, purinergic signaling is the second major contributor to detrusor mass contraction. Atropine-resistant, ATP-mediated detrusor contractions have been shown to increase with age [42]. Histamine can evoke calcium transients in cultured human detrusor smooth muscle cells [43], and histamine receptors have been the target of IC therapy with H1 [44] or H2 [45] selective antihistaminics. Therefore, we routinely analyze the expression of muscarinic (M2, M3), purinergic (P2X1, P2X2, P2X3), and histamine (H1, H2) receptors in the detrusor smooth muscle cells by confocal immunofluorescence. In double immunolabeling with alpha-smooth muscle cell actin (aSMCA), which is located directly beneath the cellular membrane of detrusor myocytes, quantification of receptor immunofluorescence could be restricted to receptors located in cell membrane. Individual receptor expression profiles are generated (Figure 3), and based on those, we developed a tailored therapy concept.

The use of routine formalin-fixed bladder tissue has the advantage that there is no need for sophisticated probe preparation and retrospective studies can be conducted on archive material.

The concept of tailored therapy based on molecular diagnostics has already been established for other disease entities, for example, colon carcinoma [46], and is a most promising approach in cancer management [47].

We also used quantitative real-time PCR (qPCR) to address receptor gene expression. However, we found no correlation between qPCR and protein expression (data not shown), which is in agreement with the literature [4, 48].

**Figure 4:**
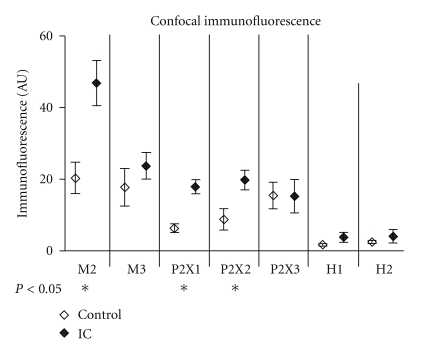
Comparison of the receptor expression in detrusor biopsies from age-matched patient collectives (control *n* = 9; BPS/IC *n* = 19). *P* values < 0.05 were considered significant (Mann-Whitney nonparametric statistical test).

**Figure 5:**
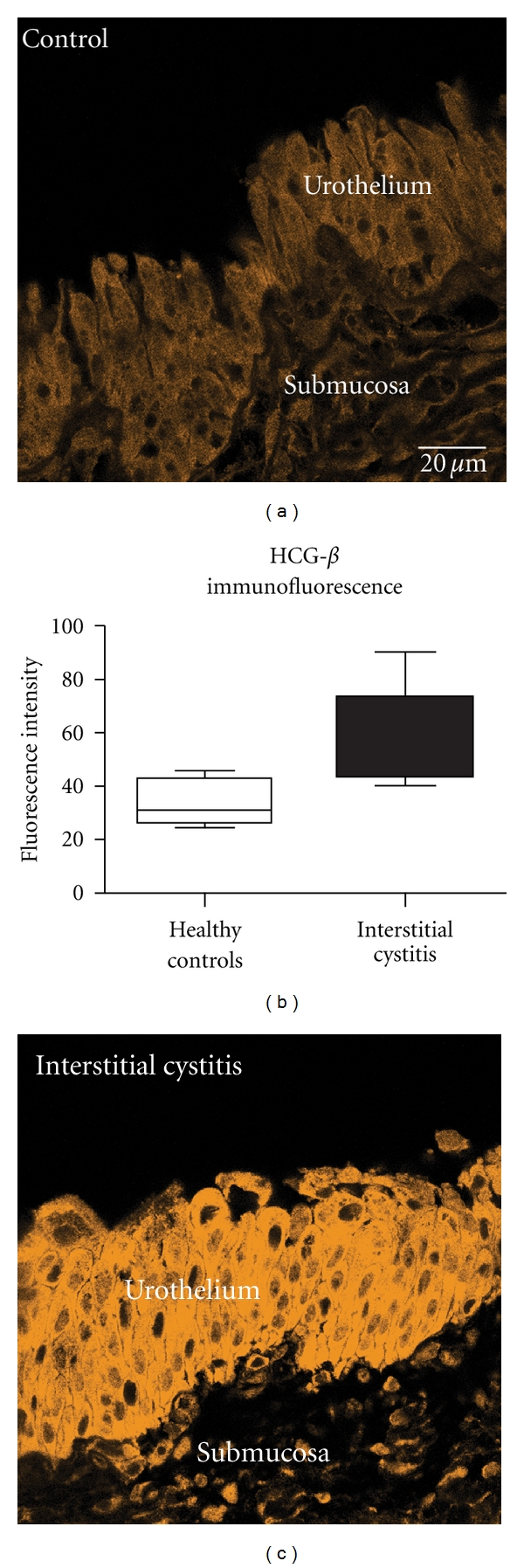
HCG-beta (beta-2 epitope) immunoreactivity in urothelium. (a) Control bladder from a 36-year-old woman, gynecologic tumor, no bladder carcinoma; (c) BPS/IC bladder of a 53-year-old woman showing extreme upregulation of HCG-beta; bar in (a) applies to (a and b); (c) Quantitative analysis revealed significant differences between female controls (*n* = 5) and female BPS/IC (*n* = 10) patients (Mann-Whitney test, *P* < 0.05).

1. *BPS/IC Patients Show a Distinct Detrusor Muscle Receptor Pattern*. Confocal immunofluorescence-based receptor profiling revealed distinct upregulation of muscarinic (M2) and purinergic (P2X1, P2X2) receptors in BPS/IC patients compared to the control group (Figure 4).
2. *Muscarinic Receptors*. BPS/IC bladders showed significant upregulation of muscarinic M2 receptor (*P* = 0.0105, Mann-Whitney test), which was also significantly higher than the M3 receptor in the detrusor of those patients (*P* = 0.0021, Wilcoxon signed rank test). In contrast, control detrusor showed equal expression of M2 and M3 receptors. In a small fraction of patients (3/19 patients, 16%), the M3 receptor expression was significantly higher than that of M2 muscarinic receptor. Numerous studies have shown that M3 selective anticholinergics are not superior to nonselective anticholinergic in respect to reduction of OAB symptoms. We suppose that this might in part be due to heterogeneity in muscarinic receptor expression in the patients collectives examined. Preselection of patients by their M2/M3 receptor expression status might, therefore, improve anticholinergic therapy.
3. *Purinergic Receptors*. Purinergic P2X receptors are expressed on detrusor smooth muscle cells and mediate atropine-resistant, ATP-evoked detrusor contractions [49, 50]. Unfortunately, to date, subtype selective antipurinergic drugs are not available for therapy. However, patients with overexpression of P2X receptors showed good response to botulinum-toxin A (BoNT-A) injection therapy. BoNT-A is thought to inhibit both efferent motor nerves and afferent sensory nerve [51]. Our own preliminary studies of BoNT-A effect on the receptor expression in the bladder speak in favor of a modulation of receptor expression in detrusor smooth muscle cells (preliminary report [52]).
4. *Histamine Receptors*. In our collective, both H1 and H2 histamine receptor subtypes were slightly upregulated (Figure 4), however, without reaching significance level. The expression showed high individual variability (H1 > H2 (6); H1 = H2 (10); H2 > H1 (3)) and 3 patients showed exceptionally high histamine receptor expression in the range of M3 or purinergic receptor expression. Antihistaminic therapy using H1-selective or H2-selective antihistaminic drugs was based on the finding of enhanced mast cell infiltration in IC patients [53]. Unfortunately, the outcome of the studies varied considerably. Therefore, antihistaminic therapy has not been established widely as therapeutic option. Histamine receptors are expressed in detrusor smooth muscle cells and might be highly overexpressed in individual cases. Histamine can evoke calcium transients in cultured human bladder smooth muscle cells [43, 54] and detrusor contractions [55]. Therefore, antihistaminics may act directly on detrusor smooth muscle cells. The heterogeneity of histamine receptor expression may well account for the high variability of therapeutical success. In case of upregulation, H1 or rather H2 antagonists would promise maximal effect of antihistaminic therapy, which might be used in combination with receptor expression-adapted anticholinergics.
5. *Molecular Diagnostics of the Bladder Urothelium*. BPS/IC is a disease of the complete bladder wall, including detrusor, submucosa, and urothelium. Therefore, it is essential to include pathology of the different cellular components into BPS/IC differential diagnosis. Despite pathology of the urothelium are most prominent in cystoscopic examination and breakdown of the urothelial urine-tissue barrier is well recognized as major pathophysiological factor in BPS/IC, cellular alterations have not been investigated intensively. Based on our own clinical experience, we examined the expression of human chorionic gonadotropin beta (HCG-beta) in the urothelium of BPS/IC and control patients. An interesting, still not explainable phenomenon is the clinical observation that IC symptoms ameliorate in female patients during pregnancy or infertility treatment with (HCG-beta). We found expression of HCG-beta in the urothelium throughout the urinary tract. Interestingly, HCG-beta is expressed in females and males, which indicates a new, unknown function of this hormone. HCG-beta can no longer be regarded as pregnancy-related hormone or tumor marker, since meanwhile our research group found constitutive expression of HCG-beta in bowel and eye (unpublished data). Two distinct HCG-beta isoforms, which are coded by different genes: type 1 (HCG-beta 6,7) and type 2 (HCG-beta 3,5,8) are differentially expressed throughout the body [56]. While type 2 is expressed in placenta and in malignant tumors, type 1 is expressed in nontrophoblast tissues. It is especially interesting that the endometrial production of HCG-beta varies in the female during normal menstrual cycle, reaching maximal concentrations in the late secretion phase [57, 58]. The effect of HCG-beta in the endometrium includes cell differentiation and neovascularization and might serve as a model for the restoration of urothelial lining in BPS/IC. In a preliminary study, we found upregulation of HCG-beta in the urothelium of nonpregnant BPS/IC women and also in men (Figure 5). Restoration of the destructed urothelial barrier in BPS/IC could be promoted by HCG-beta therapy and would be a causal therapeutical concept.

## 4. Conclusions

BPS/IC is a complex bladder dysfunction involving all cellular layers of the bladder. Current medicinal therapies lack consistent success. We propose a diagnostical concept including cystoscopic examination, histopathological evaluation, and the assessment of neurotransmitter expression profile to develop a tailored BPS/IC therapy.

Based on the expression levels of muscarinic receptors, it seems likely that patients with M3 receptor overexpression would profit most from M3 selective anticholinergics, while unselective anticholinergics may be the better choice for patients showing high levels of M2 receptors, as found in the majority of BPS/IC patients in our study. In case of significant histamine receptor expression, subtype selective antihistaminic therapy should be tried. Patients resistant to anticholinergic therapy might also profit from BoNT-A injection therapy, which might be a good choice especially if purinergic receptors are overexpressed. We further propose the evaluation of human chorionic gonadotropin expression in urothelial cells, which might lead to new therapeutical options for BPS/IC treatment.

To date, it seems likely that BPS/IC patients would profit most from combination of various receptor inhibitors adapted to their individual receptor profile. In addition, urothelial regeneration could be the clue to long-lasting success in BPS/IC therapy. There is a urgent need for clinical studies to verify the benefit of tailored therapy concept proposed here.

## Figures and Tables

**Table:** (a) Primary antibodies

Primary antibodies	Host	Source	Order no.	Dilution
M2, muscarinic receptor	rabbit	[1]	AS-3721S	1 : 1000
M3, muscarinic receptor	rabbit	[1]	AS-3741S	1 : 1000
P2X1, purinergic receptor	rabbit	[2]	ab10248	1 : 1000
P2X2, purinergic receptor	rabbit	[2]	ab10266	1 : 1000
P2X3, purinergic receptor	rabbit	[2]	ab10269	1 : 1000
H1, human histamine receptor 1	rabbit	[3]	H1R12-A	1 : 250
H2, human histamine receptor 2	rabbit	[3]	H2R22-A	1 : 250
HCG (beta-1 epitope)	mouse, IgG1	[5]	MCA19	1 : 500
HCG (beta-2 epitope)	mouse, IgG1	[5]	MCA329	1 : 20
alpha-smooth muscle cell actin	mouse, IgG2a	[4]	A2547	1 : 2000

**Table:** (b) Secondary antibodies

Secondary antibodies	Source	Order no.	Dilution
Alexa Fluor 488 goat antimouse IgG2a	invitrogen	A-21131	1 : 500
Alexa Fluor 555 goat antirabbit	invitrogen	A-21428	1 : 500
Alexa Fluor 555 goat antimouse	invitrogen	A-21127	1 : 500

[1] Research & Diagnostic Antibodies, North Las Vegas, USA.

[2] Abcam Inc., Cambridge, USA.

[3] Alpha Diagnostic Intl. Inc., San Antonio, USA.

[4] Sigma-Aldrich Chemie GmbH, Steinheim, Germany.

[5] AbD Serotec, MorphoSys AG, Martinsried/Planegg, Germany.

**Table 2:** Primers used for real-time PCR.

Primer	Sequence 5′ → 3′	Product length (bp)	Binding site	AccNo
h36B4 forward	AACATGCTCAACATCTCCCC	397	exon 6	NR_002775.1
h36B4 reverse	CCGACTCCTCCGACTCTTC		exon 8	
aSMCA forward	CCAACTGGGACGACATGGAAA	212	exon 4	NM_001613.2
aSMCA reverse	GCGTCCAGAGGCATAGAGAGACA		exon 6	
M2 forward	CTAAGCAAACATGCATCAGAATTGG	288	exon 6	NM_001006632.1
M2 reverse	AAGGTGCACAAAAGGTGTTAATGAG		exon 6	
M3 forward	ACCCAGCTCCGAGCAGATGGAC	341	exon 5	NM_000740.2
M3 reverse	CGGCTGACTCTAGCTGGATGGG		exon 5	
P2×1 forward	GCGTAATAAGAAGGTGGGCGTTA	109	exon 1	NM_002558.2
P2×1 reverse	GCCGCTCGAGGTCTGGTA		exon 2	
P2×2 forward	CAGGTTTGCCAAATACTACAAGATCA	105	exon 8	NM_174873.1
P2×2 reverse	AACTTCCCGGCCTGTCCAT		exon 9	
P2×3 forward	TCTTCACCTATGAGACCACCAAGTC	83	exon 1	NM_002559
P2×3 reverse	GATCAGAAGCTGAACTACTCGGTTGAT		exon 1	
H1 forward	AAGTCACCATCCCAAACCCCCAAG	151	exon 3	NM_001098213
H1 reverse	TCAGGCCCTGCTCATCTGTCTTGA		exon 3	
H2 forward	AGGAACGAGACCAGCAAGGGCAAT	198	exon 2a	NM_022304
H2 reverse	GGTGGCTGCCTTCCAGGAGCTAAT		exon 2a	

h36B4 = human acidic ribosomal protein P0; aSMCA = alpha-smooth muscle cell actin.
